# Lessons learned in developing family medicine residency training programs in Japan

**DOI:** 10.1186/1472-6920-5-33

**Published:** 2005-09-15

**Authors:** Mitsuya Murai, Kazuya Kitamura, Michael D Fetters

**Affiliations:** 1 Kashima Hospital, Kashima City, Japan; 2 Department of General Medicine, Nagoya University Hospital, Nagoya, Japan; 3 University of Michigan Medical Center, Department of Family Medicine, Ann Arbor, USA

## Abstract

**Background:**

While family medicine is not well established as a discipline in Japan, a growing number of Japanese medical schools and training hospitals have recently started *sougoushinryoubu *(general medicine departments). Some of these departments are incorporating a family medicine approach to residency training. We sought to learn from family medicine pioneers of these programs lessons for developing residency training.

**Methods:**

This qualitative project utilized a long interview research design. Questions focused on four topics: 1) circumstances when becoming chair/faculty member; 2) approach to starting the program; 3) how Western ideas of family medicine were incorporated; and 4) future directions. We analyzed the data using immersion/crystallization to identify recurring themes. From the transcribed data, we selected representative quotations to illustrate them. We verified the findings by emailing the participants and obtaining feedback.

**Results:**

Participants included: five chairpersons, two program directors, and three faculty members. We identified five lessons: 1) few people understand the basic concepts of family medicine; 2) developing a core curriculum is difficult; 3) start with undergraduates; 4) emphasize clinical skills; and 5) train in the community.

**Conclusion:**

While organizational change is difficult, the identified lessons suggest issues that merit consideration when developing a family medicine training program. Lessons from complexity science could inform application of these insights in other countries and settings newly developing residency training.

## Background

Like many countries in the world, the discipline of family medicine is not well established in Japan. This is surprising, given an effective national health insurance system [1,2], and large number of ambulatory care physicians in Japan. In 2002, about 34.4% of physicians reported working as a solo practitioner or as an employee in a "clinic" (defined as a physician's office without beds or with fewer than 20 beds) [3]. The majority of Japanese physicians receive specialty training in university hospitals or large hospitals for five to ten years, then about one third of them become private practitioners in a clinic [4]. Japanese physicians are not restricted by regulations based on their training or board certification and can label themselves by the kind of practice they wish to have [5]. By default, these sub-specialty-trained doctors become Japan's self-taught primary care doctors, but they are neither systematically trained for this field nor do they have much opportunity to gain knowledge and skills necessary for primary care through continuing medical education.

Previous authors have synthesized information on the need for family medicine in Japan and provided status reports on the development of family medicine [4-7]. While the family medicine movement in Japan began in the 1970's, the first department of general medicine was not established until 1981 at Kawasaki Medical School. The most important academic organization supporting family physicians is the Japanese Academy of Family Medicine (JAFM). Established in 1986, it has taken a leadership role in establishing family medicine in Japan. Like most medical societies in Japan, the JAFM has membership based on interest, not criteria such as board certification or completion of specific residency training. As family medicine is still a young discipline in Japan, most members have trained in non-family medicine programs. The physician members include a diverse group: those trained in a family medicine training program in Japan or abroad (mostly the US), those trained in another specialty or multiple specialties and became a general practitioner after entering practice, and those who trained in a general internal medicine program in Japan or abroad.

A barrier to family medicine's development continues to be inconsistent support from the Japanese government. At times enthusiastic, at times apathetic about family medicine's establishment, the government has faced stiff opposition from the Japan Medical Association and previously has backed down from its own initiatives to support family medicine. Interestingly, the Japanese Ministry of Health, Labour, and Welfare (MHLW) recently approved creation of *sougoushinryoubu *(translation: general medicine departments)[8]. Of the 80 medical schools in Japan, 30 have established *sougoushinryoubu*, and 96 training hospitals have established, or are preparing, a new training program [8]. Unfortunately, the MHLW did not provide clear direction about the content and purpose of these departments, and most have developed according to one of three patterns. About ten of the medical school *sougoushinryoubu *decided to use family medicine as a model for their development. An even smaller number appear to be pursuing a general internal medicine model, and the remainder has not made a commitment to a discipline. These often function as basic triage departments to funnel patients to hospital-based sub-specialists who do not want to manage undifferentiated problems.

Given our interest in the development of family medicine in Japan, and the lack of literature describing the issues involved in starting a department of family medicine training program, we were interested in the experiences and opinions of the pioneers who are embracing development of family medicine. The objectives of this research were to investigate their experiences with developing family medicine residency training in Japan, and to draw lessons for others.

## Methods

This qualitative project utilized a long interview research design [9] since the intent was to elicit, in detail, the family medicine pioneers' experiences with, and their opinions about, the development of family medicine training programs in Japan. During a one-month research elective from his US residency training program, one of us (MM) traveled to Japan in November 2000 to conduct interviews with as many Japanese faculty "pioneers" as could be arranged. As the authors are members of the JAFM, and actively participate, we are familiar with the small number of individuals taking a leadership role in family medicine activities in the JAFM, and directly contacted potential candidates. The eligibility requirements of recruited sites included: having at least one faculty member graduated from a family medicine program in Canada or the U.S., or who had had several months of learning experiences in undergraduate/family medicine in the U.S.; and/or having at least one faculty member with broad and long experience in community-based general practice.

One of us (MM) conducted in-depth, open-ended 40 to 75 minutes duration interviews. He conducted each interview in an office, or other private setting selected by the participant. We developed the interview guide in the format proposed by Crabtree and Miller for conducting "Long Interviews." [9]. The interviews began with a "grand tour" style question: "Think back and describe the circumstances that lead up to your becoming the chair/faculty member of the program." This was followed with a series of probe questions (see Appendix 1). Interview questions focused on four topics: 1) circumstances when becoming chair/faculty member; 2) approach to starting the program; 3) how Western ideas of family medicine/general medicine were incorporated; and 4) future directions. We asked participants about unanticipated issues that they had encountered. Each interview was audiotaped and field notes were kept in a study journal. The interviews were transcribed by the native Japanese-speaking researchers and a research assistant, who were instructed to record verbatim the conversation including pauses, repetitions, etc. [10].

For the content analysis, we utilized the techniques of immersion/crystallization [11,12]. The primary analysis team (MM and KK) conducted multiple readings of the transcripts and independently identified the major themes from the transcribed text. Both analysts found the participants discussed lessons they had learned, and barriers and facilitators, to development of family medicine. Within these areas, they found five overarching lessons to organize the primary findings. To ascertain these were grounded in the interviews, they searched the transcribed text to identify examples corroborating the findings.

After organization into a narrative format, we sent these results to the participants by email, and requested their comments on our interpretations, and specifically on the issue of economics of starting a training program as this area had been discussed little during the interviews. There were no substantive changes based on the feedback.

## Results

The participants were ten Japanese faculty leaders of family medicine programs: two program directors, five chairpersons, and three faculty members. The facilities where they work include two private medical colleges, two private community hospitals, and three public universities. The participants included nine male, and one female, physicians.

Based on our analysis of the text and field notes, we identified five themes, two highlighting the barriers and three signifying facilitators for developing family medicine training. We organized them as five lessons: 1) "They just don't get it;" 2) developing a core curriculum is difficult; 3) start with undergraduates; 4) emphasize clinical skills; and 5) train in the community.

### Lesson 1: They just don't get it

In the environments where these faculty members work, few of their colleagues understand the basic concepts and values of family medicine. In their experience, high-level administrators, CEOs, deans, and hospital directors, are the most supportive of new departments. For example, the latter group supports dispatching eligible faculty members to foreign countries to learn family medicine and to recruit board-certified family physicians, or experienced community-based general practitioners, before starting a new department. These administrators have helped resolve conflicts between the fledgling department and other specialty departments.

However, such administrators have not supported the opening of community-based family medicine teaching clinics, as they do not appreciate the need. The interviewed faculty also feel that the high-level administrators do not advocate the distinguishing features and the objectives of family medicine to faculty. These pioneers continue to work in a hostile environment alongside faculty from other departments that lack an understanding of family medicine. One participant stated, "Current Japanese specialists make no sense, saying [they practice] primary care cardiology etc." Another noted, "Faculty members of other departments with five to ten years of experience verbally harass family medicine applicants/residents with comments, such as, 'Family Medicine? Training in ambulatory care? Nonsense! Out of the question! Quit that department!' "

Even some residents in family medicine-oriented departments "don't get it" as they may resist rotations that faculty view as core, such as pediatrics, OB/GYN, or behavioral science. Their reluctance highlights a gap in the residents' intellectual understanding of family medicine and their inability to grasp the value of rotating in other departments to see patients and problems needed to acquire appropriate knowledge and skills to be a family physician. This also hinders designing a core curriculum.

### Lesson 2: Developing a core curriculum is difficult

These leaders find developing a core curriculum to be difficult for three reasons: 1) residents are laborers first, learners second; 2) disinterest of residents in certain core rotations; and 3) lack of a critical mass of residents to ensure coverage for other services.

Hospital-based specialties need a work force for clinical care in their departments. The lack of work hour restrictions in Japan emphasizes the view of residents as laborers. Residents commonly "rotate" among various clinical services that are hospital-based and demand a 24-hour per day commitment to patient care by the team. Even senior family medicine residents cannot leave other rotations and return for a half-day of clinic. One participant stated, "I can't ask other departments to teach knowledge and skills relevant to primary care because rotating residents are their work-force." Residents feel pressured not to leave their rotations in other departments saying, "I feel uncomfortable leaving the rotating department to come back for a half-day clinic."

As in Lesson 1, participating faculty note that residents are sometimes opposed to certain "core" family medicine rotations. Rather, residents request elective rotations that they think will be interesting. Faculty must take these requests seriously, since the number of residents in any given year can fluctuate widely depending on graduating medical student interest and the departments are competing for these graduates. This variation in interest makes negotiation of demands about content and return to clinic while on other specialty rotations services challenging. The family medicine programs need a critical mass of residents for core clinical services, but ideally could supply a consistent number of family medicine residents for other departments.

These conflicting demands make it difficult for most programs to have a standardized residency system. One participant stated, "We don't have a national matching system like in America. It is unpredictable how many students [will] enter the program next year. Maybe five, maybe zero. In such circumstances, we are unable to provide resident availability either in [our] home or in other departments. Therefore, it is next to impossible to establish a curriculum with core rotations and elective rotations. We can't help but approve elective rotations that are simply interesting to residents. We can't stop drop-out from the program."

### Lesson 3: Start with undergraduates

Five participants indicated that it is too late to start exposure to family medicine at the resident level. Residents have pre-conceived notions about the content of clinical practice. One participant stated, "Some residents declare that they are going to practice internal medicine, so they do not need pediatrics, OB/GYN, or behavioral science etc. After they graduate, they send me a letter [saying] they now realize they lack training in these areas, ha ha ha." Another criticized, "If I could teach [students] the basic concepts and the value of family medicine in 10–15 hours at the end of medical school education, [after] four years of brain-washing by "specialists," family medicine could have an impact on medical students." Many of these family medicine leaders need and want to be more involved in undergraduate curriculum reform.

### Lesson 4: Emphasize clinical skills

Currently, the medical school curriculum is not structured to effectively teach clinical skills. One participant stated, "Many Japanese students are unable even to measure blood pressure, to take a medical history, and to perform a physical examination at [the time of] graduation. They aren't competent because they were not taught. Our department proved it." Medical students are hungry for basic clinical skills, such as medical interviewing and performing the physical examination; and family physicians excel at these skills. The feedback from students in programs where these skills are emphasized is uniformly positive.

As individuals, faculty members of medical school and training hospitals sometimes lack effective teaching skills. One participant stated, "They don't know the principles of adult education, nor how to teach clinical medicine. Their belief is that knowledge and skills are 'stolen' from senior residents or attending physicians, not taught." Another lamented, "There are few faculty development curricula in Japan." It is an area needing development in Japanese family medicine.

### Lesson 5: Train in the community

Collectively, these faculty felt training in community-based outpatient sites has been most effective. One participant stated, "Our department started in the early 1980's. The first seven years were chaotic. There were a lot of conflicts and confusion within the department. After I took over and oriented [the department] towards family medicine in '89 most things went well, especially after opening a model office in the community." Another participant stated, "I trained residents in the community since the beginning."

University hospital-based sites have been less effective due to the poor relationship with other departments and the difficulty of being a "role model" in the tertiary care center. Specific reasons for the latter include selection bias of the patients and poor continuity of care. A faculty member states, "There is no way to solve the problem of continuity other than going to the community."

## Discussion

These Japanese leaders' experiences echo many of the stories heard in the history halls of family medicine departments and residency programs in other countries with more established family medicine training. Institutional structure, politics, and national policy have a significant impact on the establishment and growth of family medicine in the culture of academic medicine in Japan. While previous literature identifies resident perspectives,[13] these lessons from faculty provide insights into issues that will likely be encountered in starting family medicine programs in, and outside, of Japan as well. While their relative importance may be different, it is likely that the issues identified here will have some universality in terms of developing a family medicine program anywhere.

Efforts to educate others about the values and content of family medicine are strongly needed. Other departments may fail to understand family medicine since it is a horizontal specialty which cuts across the lines of existing specialties [14]. Moreover, it is defined more by a value system rather than a unique body of knowledge [15]. Harvey pointed out in 1985, "There are deep philosophical differences between the traditional clinical departments and divisions... They do not understand the emphasis on the family, the educational principles, and many, many other matters of importance to family medicine programs." [16]. This may help explain the superficial support of high-level administrators.

Fledgling departments of Family Medicine around the world must make their residency training goals clear to partnering departments and their residents. Residents are a work force. Too often, training is focused on inpatient care and taught by "specialists" in tertiary care centers or large training hospitals without an understanding of the purpose of the rotation for the resident. The faculty members and senior residents in other specialty departments do not know "what" and "how" to teach family medicine residents [17,18]. As indicated by Doherty, the timing, and duration of exposure to specialists merits careful attention [19]. He states, "...most family medicine experiences in outpatient and inpatient care tend to be postponed until after internship, thereby complicating the "imprinting" experiences of young physicians in training. Thus the delicate socialization of the family physician toward a role that is neither organ-system nor technology-specific is largely in the hands of non-family physicians, who by definition cannot model the unique identity of the family physician. Next generations of family practice residents suffer from the same identity confusion as its elders (p. xiv)."[19]

A balance between hospital-based and ambulatory-based training is needed. University or tertiary care center training is important for nurturing logical approaches to post-graduate education, research, and unsolved problems [17]. On the other hand, busy practitioners have broad practical knowledge and experience in treating patients. Both offer benefits to family medicine students and residents. Training programs need to, and can develop bridges with community-based physicians [18].

Critically important to family medicine teaching is a model family medicine office as a clinical classroom for training the resident and fostering her/his identity as a family physician [20]. Japanese pioneers face significant financial and political barriers including opposition from the Japan Medical Association and ambiguous support from the government [20]. While it is possible to open a resident teaching site at the beginning of the residency program, [19] a university-affiliated clinic may be perceived as a threat to practitioners and face stiff opposition from the local medical society. Suffice it to say that the specific political barriers are likely to differ from country to country and locale to locale, but pioneers of new programs will most assuredly encounter political barriers.

Based on the experiences of these participants, future family medicine program developers can anticipate benefits from early exposure of family medicine to medical students in the undergraduate curriculum, and emphasizing teaching skills that their students, as aspiring physicians, are driven to learn. Strongly tied to this is the need to focus on faculty development.

A growing literature illustrates the relevance of complexity science to understand family practice offices as complex adaptive systems [21,22]. As would be predicted by complexity science, organizational change will be difficult given the interactions and interdependencies that exist and must evolve for new departments to grow [23-26]. For example, newly developing departments of family medicine must be imbedded within the overall hospital and university systems and will require their "buy-in" [23]. The "five lessons" here might be considered "minimum specifications"[24] for guiding development of new family medicine departments with an expectation that there would be "wide space for innovation and shared action" in each new site. Research by Anderson and colleagues provide just one illustration of how the application of complexity science can enhance care quality [27]. We believe pioneers of family medicine departments would be wise to apply the conceptual aspects of complexity science to their specific situations as a framework for organizing development efforts, and perhaps even into their teaching approaches [28].

The potential limitations of this research are selection bias and a small sample size. However, the sample is close to the population of eligible family medicine pioneers. Only one woman participated, although this level of representation is close to the population of female physicians (about 14 %) in Japan [3]. Our data collection procedure was primarily limited to individual interviews. In future research, using multiple data collection techniques[22,29,30] could facilitate through triangulation a more detailed examination of successful programs, and provide further insight into developing family medicine residency training programs. In addition, a case comparison between Japan and Korea, an Asian country that has embraced family medicine, could further illustrate the importance of social, cultural, economic, and political differences that influence development of family medicine as a discipline. Finally, additional reports about experiences from other countries and contexts could extend the dialogue about issues salient to developing family medicine training programs.

## Conclusion

While organizational change is difficult, the identified lessons suggest issues that merit consideration when developing a family medicine training program. We conclude that developing family medicine departments should: make residency training goals clear to their residents and partnering departments; balance hospital-based and community-based training; develop a model family medicine office as a clinical classroom; and actively participate in medical student education. Lessons from complexity science could inform application of these insights in other countries and settings newly developing residency training.

## Abbreviations

JAFM-Japanese Academy of Family Medicine

MHLW-Ministry of Health, Labour, and Welfare

## Competing interests

The author(s) declare that they have no competing interests.

## Authors' contributions

MM conceived the study, participated in the research design, conducting the interviews and transcribing them, the primary qualitative analysis, and manuscript writing. KK participated in the primary qualitative analysis and manuscript writing. MF participated in the design, coordination, confirmatory qualitative analysis, writing and revision of the manuscript. All authors read and approved the final manuscript.

## Pre-publication history

The pre-publication history for this paper can be accessed here:


